# Novel application of an automated-machine learning development tool for predicting burn sepsis: proof of concept

**DOI:** 10.1038/s41598-020-69433-w

**Published:** 2020-07-23

**Authors:** Nam K. Tran, Samer Albahra, Tam N. Pham, James H. Holmes, David Greenhalgh, Tina L. Palmieri, Jeffery Wajda, Hooman H. Rashidi

**Affiliations:** 1 Department of Pathology and Laboratory Medicine, University of California Davis, 4400 V St., Sacramento, CA 95817 USA; 2 Department of Surgery, University of Washington, Washington, USA; 3 Department of Surgery, Wake Forest University, Winston-Salem, USA; 4 Department of Surgery, UC Davis, Davis, USA; 5 UC Davis Health, Davis, USA

**Keywords:** Infectious diseases, Trauma, Scientific data, Software

## Abstract

Sepsis is the primary cause of burn-related mortality and morbidity. Traditional indicators of sepsis exhibit poor performance when used in this unique population due to their underlying hypermetabolic and inflammatory response following burn injury. To address this challenge, we developed the Machine Intelligence Learning Optimizer (MILO), an automated machine learning (ML) platform, to automatically produce ML models for predicting burn sepsis. We conducted a retrospective analysis of 211 adult patients (age ≥ 18 years) with severe burn injury (≥ 20% total body surface area) to generate training and test datasets for ML applications. The MILO approach was compared against an exhaustive “non-automated” ML approach as well as standard statistical methods. For this study, traditional multivariate logistic regression (LR) identified seven predictors of burn sepsis when controlled for age and burn size (OR 2.8, 95% CI 1.99–4.04, P = 0.032). The area under the ROC (ROC-AUC) when using these seven predictors was 0.88. Next, the non-automated ML approach produced an optimal model based on LR using 16 out of the 23 features from the study dataset. Model accuracy was 86% with ROC-AUC of 0.96. In contrast, MILO identified a *k*-nearest neighbor-based model using only five features to be the best performer with an accuracy of 90% and a ROC-AUC of 0.96. Machine learning augments burn sepsis prediction. MILO identified models more quickly, with less required features, and found to be analytically superior to traditional ML approaches. Future studies are needed to clinically validate the performance of MILO-derived ML models for sepsis prediction.

## Introduction

Burn patients are at high risk for infections, with sepsis being the most common cause of morbidity and mortality^1^. Traditional indicators of sepsis defined previously by the Surviving Sepsis Campaign^2^ and other organizations exhibit poor performance when used in this unique population due to their underlying hypermetabolic and inflammatory response to burn injury. For example, the systemic inflammatory response syndrome^2,3^ lacks clinical sensitivity and specificity when applied to severely burned patients^1^, while the newer 2016 “Sepsis-3” criteria remain controversial in both burned and non-burned patients^4–7^. To this end, early and accurate recognition of sepsis represents a significant clinical knowledge gap in burn critical care.

The American Burn Association (ABA) Consensus Guidelines published in 2007 was intended to better differentiate burn sepsis from the natural host-response to injury (Table 1)^1^. These guidelines recognized deficiencies of traditional indications of sepsis and removed less specific parameters such as white blood cell count (WBC). Fever was re-defined as temperatures > 39 °C to improve specificity and at the cost of sensitivity. Glycemic variability and thrombocytopenia were also included in the ABA Consensus Guidelines; however, measurement of glycemic variability is challenging without continuous glucose monitoring technology and platelet count aids burn sepsis recognition at later stages of severe infection.

**Table 1:**
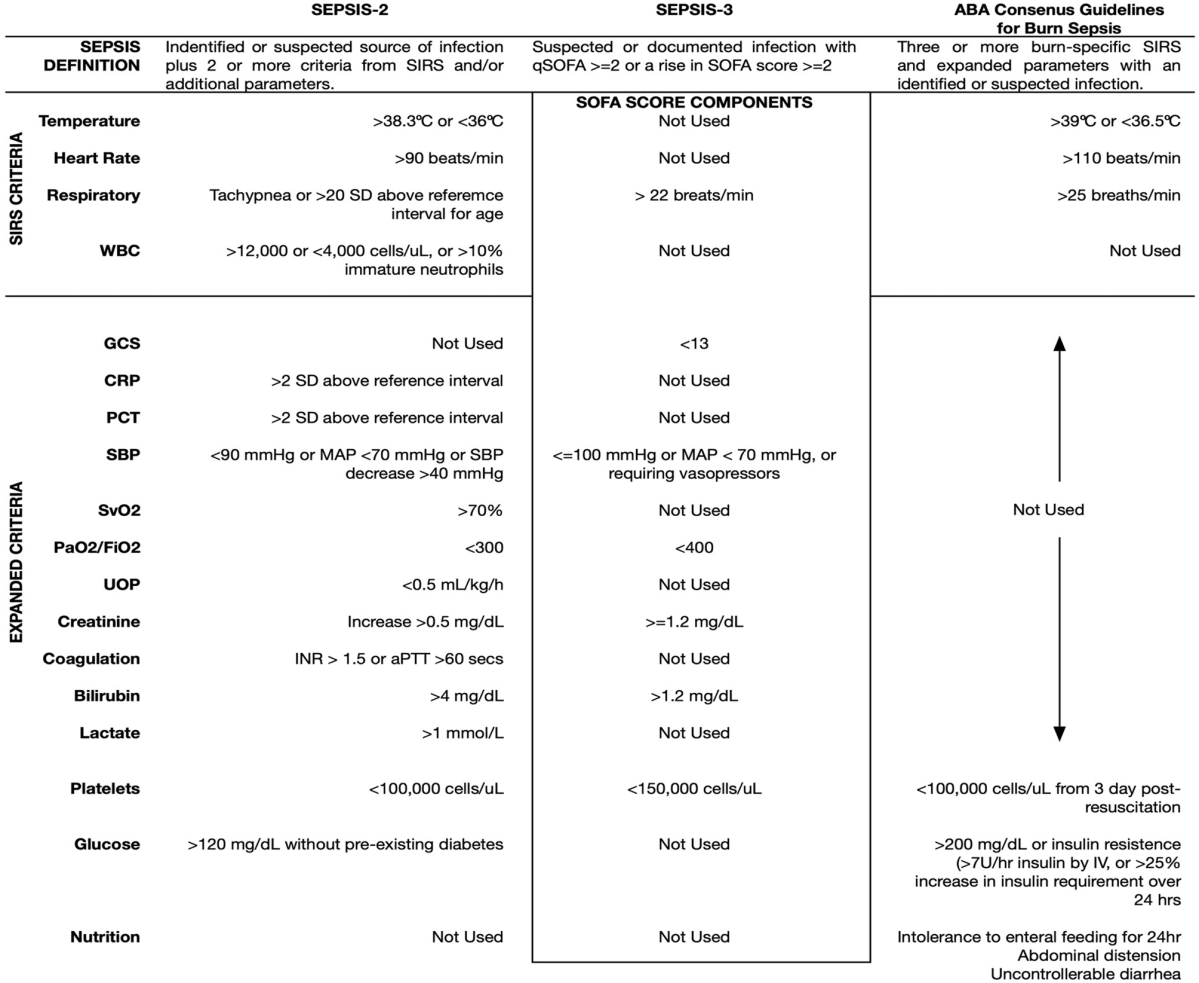
Comparison of sepsis criteria.

*ABA* American Burn Association, *aPTT* activated partial thromboplastin time, *CRP* c-reactive protein, *FiO2* fraction of inspired oxygen, *GCS* Glascow Coma Score, *INR* international normalized ratio, *IV* intravenous, *NS* not significant, *PaO2* partial pressure of oxygen, *PCT* procalcitonin, *PLT* platelet count, *SBP* systolic blood pressure, *SD* standard deviation, *SIRS* systemic inflammatory response syndrome, *SOFA* sequential organ failure assessment, *SvO2* saturation of venous oxygen, temp temperature, *UOP* urine output, *WBC* white blood cell count.

The emergence of artificial intelligence (AI) and machine learning (ML) provides an opportunity to improve burn sepsis recognition. Recent studies suggest AI/ML improves the diagnostic accuracy, and clinical sensitivity/specificity for predicting burn related sequalae such as acute kidney injury (AKI)^8^. However, widespread adoption of AI/ML in laboratory diagnostics is challenged by the lack of programming expertise in the medical community and accessibility of health data to develop clinically relevant models^9^. To this end, the development of automated ML platforms to facilitate pragmatic clinical studies are needed to fully realize the true capabilities of health AI. The objective of this study is to provide proof of concept clinical utility of ML for sepsis recognition in comparison to existing criteria in the high-risk burn population.

## Methods

We developed and validated ML models for burn sepsis prediction using a retrospective dataset. The database was derived from a previous multicenter (5-site) randomized controlled trial (ClinicalTrials.gov#NCT01140269) evaluating the clinical impact of molecular pathogen detection in burn sepsis patients where vital signs and laboratory data was recorded daily for the duration of each patient’s intensive care unit (ICU) stay (Supplemental Data Fig. S1)^10^. Vital signs and laboratory data was consistently collected per study protocol. Contributing sites were ABA verified burn centers in the United States. Human subjects’ approval was obtained at each study site and through the United States Army Human Research Protection Office. From this study population, we used the data to help predict sepsis when analyzed using traditional statistics, as well as ML using traditional non-automated programming, and then compared against our novel automated ML approach. Study methods are described below.

### Study population

The database consisted of 211 adult (age ≥ 18 years) patients with ≥ 20% total body surface area (TBSA) burns enrolled from across five United States academic hospitals. Patients with non-survivable injuries or lacking the ability to provide informed/surrogate consent were excluded. Relevant medical information including patient demographics, vital signs (i.e., heart rate, respiratory rate, systolic/diastolic blood pressure, mean arterial pressure, central venous pressure), laboratory results (i.e., blood gas indices, complete blood count, chemistry panels, coagulation status, and microbiology results), Glascow Coma Score (GCS), medical/surgical procedures (e.g., surgery, intravascular line placement/removal), mechanical ventilator settings, and prescribed antimicrobial medications were recorded daily over the course of their ICU stay. Outcome measures include sepsis status, and mortality were also recorded. Sepsis status was based on the 2007 ABA Consensus Guidelines^1^. The study population included patients with respiratory, urinary tract, soft tissue, and/or bloodstream infections. Recorded data variables are outlined in Table 2 and were used for curating the data to determine sepsis status, as well as for performing traditional statistical analyses, and ML model development and generalization.

**Table 2:** Daily recorded variables for enrolled subjects.

Vital sign	Blood gas	Chemistry	Heme/Coag	Microbiology	Calculated values	Clinical events
Heart rate	pH	Na+	HGB	Blood culture	Anion gap	Surgery
Respiratory rate	pCO2	K+	HCT	Respiratory culture	BUN/creatinine ratio	Ventilatory status
SBP/DBP	pO2	Cl−	WBC	Urine culture	MODS	Antibiotic therapy
CVP	HCO3−	TCO2	Platelet count	Wound culture	SOFA	Dialysis status
MAP	FiO2	Glucose	aPTT		PaO2/FiO2	Survival status
GCS		Creatinine	INR			
		BUN	D-dimer			
		Total bilirubin				

*aPTT* activated partial thromboplastin time, *BUN* blood urea nitrogen, *CVP* central venous pressure, *DBP* diastolic blood pressure, *FiO2* fraction of inspired oxygen, *HCT* hematocrit, *HGB* hemoglobin, *INR* international normalized ratio, *MAP* mean arterial pressure, *MODS* multiple organ dysfunction score, *PaO2* partial pressure of arterial oxygen, *pCO2* partial pressure of CO2, *pO2* partial pressure of oxygen, *SBP* systolic blood pressure, *SOFA* sequential organ failure assessment score, *SO2* oxygen saturation, *TCO2* total CO2, *WBC* white blood cell count.

### Traditional machine learning method

Machine learning sepsis algorithms were first developed using our exhaustive “traditional” non-automated ML approach^8,11^. The process entailed manually selecting various feature set combinations aided with an unsupervised select percentile techniques such as ANOVA F-classification for feature selection from the original dataset followed by building a large number of models on various supervised algorithms. The five ML supervised algorithms employed for this task included: (a) logistic regression (LR), (b) *k*-nearest neighbor (*k*-NN), (c) random forest (RF), (d) support vector machine (SVM), and our multi-layer perceptron deep neural network (DNN). Scikit-Learn’s version 0.20.2 was used to construct models as in previous studies^11^. Cross validation and hyperparameter tuning studies were also performed for LR, RF, *k*-NN, SVM, and DNN methods using the Scikit-learn cross validation and grid search tools. This technique along with the grid search hyperparameter variations allowed us to develop and compare a large number (49,940) of unique models based on various feature set combinations (identified from our select percentile feature selection) within our five ML methods/algorithms. This approach enabled us to empirically assess and compare all models and to identify the best performing ML model for a given set of unique hyperparameters and feature set combinations.

### Automated ML (auto-ML) platform

**Figure 1:**
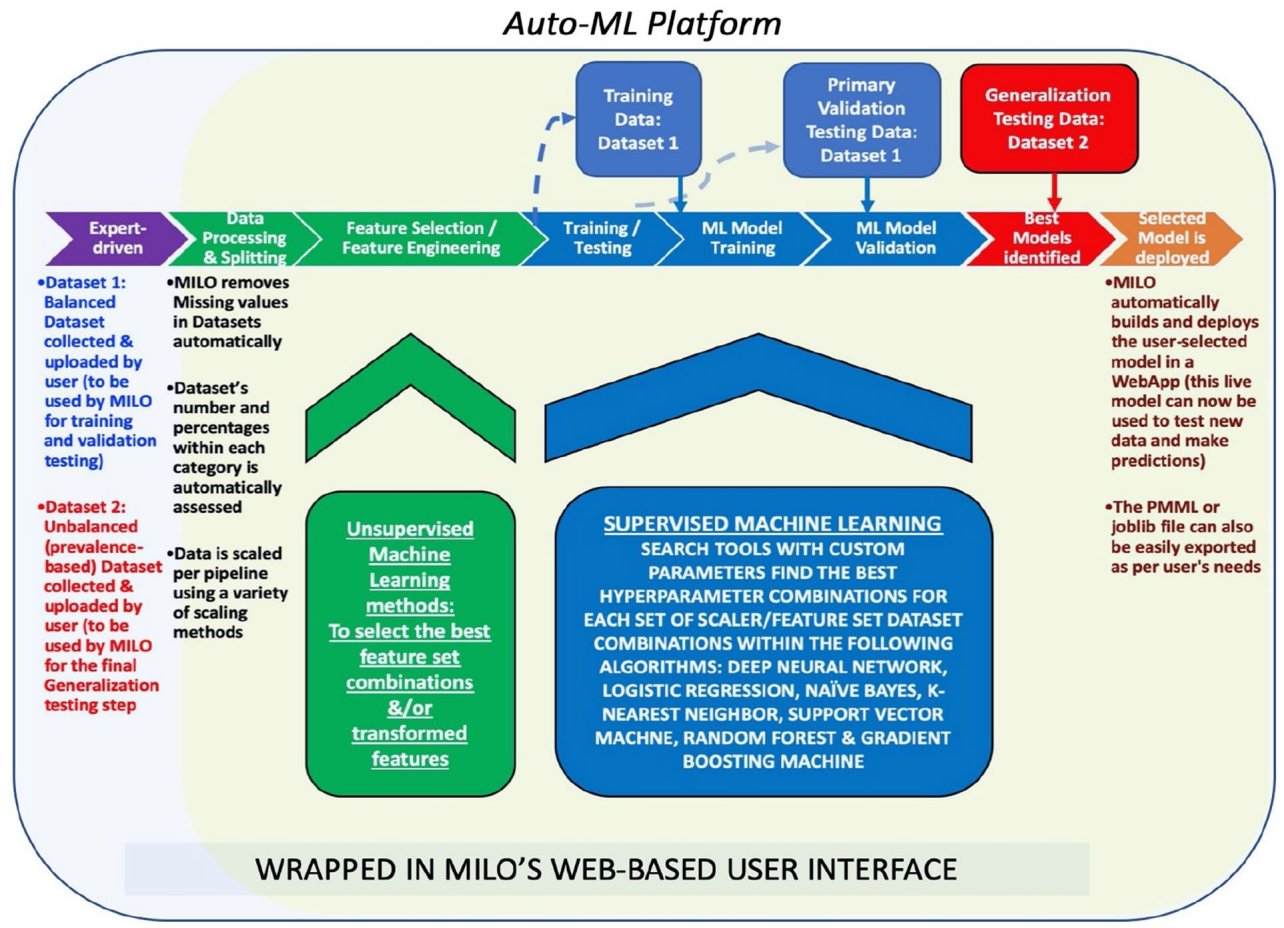
Machine intelligence learning optimizer: the MILO auto-machine learning (ML) infrastructure consists of begins with two datasets: (**a**) balanced data (Data Set 1) set used for training and validation, and (**b**) an unbalanced dataset (Data Set 2) for generalization. MILO removes missing values, assessed and scaled by the software. Unsupervised ML is then used for feature selection and engineering. The generated models are trained and then tested with the Data Set 1 during the supervised ML stage. Primary validation is then performed using Data Set 1 and followed by generalization using Data Set 2. Selected models can then be deployed thereafter as predictive model markup language (PMML) files.

In addition to the above manual ML approach, we developed the Machine Intelligence Learning Optimizer (MILO) platform to perform a similar task in a fully automated fashion (Fig. 1)^12^. The MILO infrastructure includes an automated data processor, a data feature selector (ANOVA F select percentile feature selector and RF Feature Importances Selector) and data transformer (e.g., principal component analysis), followed by custom supervised ML model building using our custom hyperparameter ranges used with search tools (i.e., grid search and random search tools) to help identify the optimal hyperparameter combinations for DNN, LR, naïve Bayes (NB), *k-*NN, SVM, RF, and XGBoost gradient boosting machine (GBM) techniques. Additionally, MILO enables addition of other algorithms and hyperparameter combinations—allowing us to easily add in NB and GBM for analysis.

Following the training and validation of models, MILO executes an automated performance assessment with results exported for user viewing. In the end, MILO employs a combination of unsupervised and supervised ML platforms from a large set of algorithms, scalers and feature selectors/transformers to create greater than 1,000 unique pipelines (i.e., set of automated machine learning steps)—ultimately generating > 100,000 models that are then statistically assessed to identify optimal algorithms for use (Supplemental Data Table S1).

For this study, we imported the trial data into MILO using sepsis status as the outcome measure for analysis. The following functions are then performed automatically by MILO. First, rows with any missing values are removed (e.g., laboratory results that were not performed for a given day). Next the information is assessed to ensure model training and the initial validation step is based on a balanced dataset. A balanced dataset is used for training because the system was built to work with small amounts of training data and since accuracy was a scoring discriminator, the measured accuracy can then be better assessed against a lower null accuracy baseline which ultimately would minimize overfitting. This balanced dataset is then split into training and validation test sets in an 80–20 split, respectively. Since many algorithms benefit from scaling, in addition to using the unscaled data, the training dataset also underwent two possible scaling transformations (i.e., standard scaler and minmax scaler). To evaluate the effect of various features within the datasets, a combination of various statistically significant feature was then selected to build new datasets with less or transformed features. The features selected in this step are derived from several well-established unsupervised ML techniques including ANOVA F-statistic value select percentile, RF Feature Importances or transformed using our principle component analysis approach^9^. A large number of ML models are then built from these datasets with optimal parameters on large number of pipelines which include a combination of various algorithms (i.e., DNN, SVM, NB, LR, *k*-NN, RF, and GBM), scalers, hyper-parameters, and feature sets. All pipelines generated by MILO undergo generalization assessment no matter their performance. For model validation, MILO creates and assesses hundreds of thousands of models. All models for each category are then identified and passed onto the next phase of the software pipeline for generalization assessment. Machine learning model performance data is then tabulated by MILO and reported as clinical sensitivity, specificity, accuracy, F1 score, receiver operator characteristic (ROC) curves, and reliability curves. Finally, to evaluate if ABA Consensus Guidelines^1^ and Sepsis-3 criteria^4^ are compatible with ML applications, MILO algorithms were generated using their respective parameters (ABA Consensus Guidelines: body temperature, respiratory rate, and heart rate, platelet count (PLT), and glucose; Sepsis-3: respiratory rate, PaO2/FiO2, GCS, PLT, MAP, and total bilirubin) and compared against models evaluating optimized features from the dataset. American Burn Association Consensus Guideline^1^ criteria such as insulin rates/resistance, intolerance to enteral feedings, abdominal distension, and uncontrollable diarrhea were not available for the study dataset.

### Traditional statistical analysis

JMP software (SAS Institute, Cary, NC) was used for statistical analysis. Descriptive statistics were calculated for patient demographics. Data was also assessed for normality using the Ryan-Joiner Test. Continuous parametric variables were analyzed using the 2-sample *t*-test, while discrete variables were compared using the non-parametric Chi-square test. As appropriate, continuous non-parametric variables, the Mann–Whitney U Test was used. Multivariate LR was used to determine predictors of sepsis with age and burn size serving as covariates with 95% confidence intervals (CI) reported. Repeated measures ANOVA was used for time series data. A *P*-value < 0.05 was considered statistically significant with ROC analysis also performed to compare sepsis biomarker performance. Bootstrapping (minimum of 2,500 bootstrap samples) via JMP software was employed to calculate 95% confidence intervals (CI) for the area under the ROC curves.

## Results

**Figure 2:**
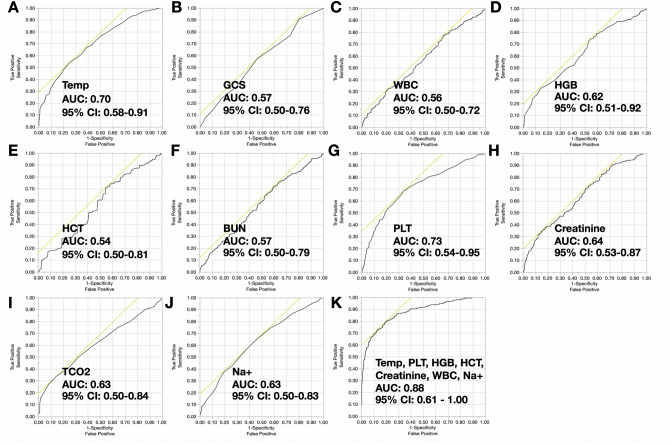
Receiver operator characteristic curves for statistically significant burn sepsis biomarkers: (**A**–**J**) represent receiver operator characteristic (ROC) curves and the area under the curve (AUC) analysis (in fractions) with 95% confidence intervals (CI) for statistically significant predictors of burn sepsis. (**K**) is the ROC curve for the multivariate model that best predicts sepsis using logistic regression. The tangent line for each ROC curve identifies the point where sensitivity and specificity are optimized. *BUN* blood urea nitrogen, *GCS* Glascow coma score, *HCT* hematocrit, *HGB* hemoglobin, *Na*+ sodium, *PLT* platelet, *TCO2* total carbon dioxide, *TCO2* total carbon dioxide.

The study dataset was compromised of data from 211 patients with 704 incidents requiring collection of cultures in suspicion of sepsis. Table 3 highlights the demographics for septic versus non-septic burn patients. Briefly, septic patients significantly differed in terms of vital signs and laboratory parameters compared to their non-septic counterparts. Multiple organ dysfunction score (MODS) (4.42 [2.8] vs. 3.87 [2.9], P = 0.006) and sequential organ failure assessment (SOFA) scores (4.10 [2.7] vs. 3.17 [2.3], P < 0.001) were also found to be significantly higher in sepsis versus non-sepsis patients. Burn sepsis patients also exhibited greater disease severity scores (maximum MODS: 17 vs. 13 and maximum SOFA: 14 vs. 10). Figure 2 compares ROC curves for these statistically significant variables. Multivariate LR identified body temperature, WBC, HGB, HCT, Na+, and PLT as predictors of sepsis when controlled for age and burn size (OR 2.8, 95% CI 1.99–4.04, P = 0.032). The area under the ROC curve when using these seven predictors was 0.88 (95% CI 0.61–1.00).

**Table 3:** Comparison of septic versus non-septic burn patients.

Variable	Septic (n = 92)	Non-septic (n = 119)	P-value
**A. Demographics**			
Mean (SD) age (years)	44.5 (18.1)	38.6 (15.7)	NS
Mean (SD) TBSA (%)	38.9 (16.9)	21.7 (10.8)	0.033
Gender (M/F)	59/33	80/39	NS
Inhalation injury (%)	14.1%	13.9%	NS
ICU LOS (days)	58.7 (25.6)	40.2 (26.2)	0.021
Median (IQR) # infections per patient			
Bloodstream	5.5 (6.0)	N/A	N/A
Pneumonia	7.2 (5.5)	N/A	N/A
Urinary tract	3.6 (2.7)	N/A	N/A
Wound	4.4 (3.6)	N/A	N/A
Mortality (%)	31.5	11.8	0.001
Mean GCS (SD)	10.3 (2.8)	11.4 (2.8)	0.005
Mean (SD) MODS	4.4 (2.8)	3.9 (2.7)	0.006
Mean (SD) SOFA	4.1 (2.7)	3.2 (2.3)	< 0.001
**B. Laboratory data**			
Median Temperature (IQR) (ºC)	39.2 (4.0)	38.0 (3.5)	0.010
Median HCT (IQR) (%)	24.0 (7.0)	25.5 (8.6)	0.003
Median HGB (IQR) (g/dL)	7.8 (2.2)	8.5 (3.0)	< 0.001
Median WBC (IQR) (cells/µL)	13.1 (10.7)	12.1 (9.0)	0.001
Median creatinine (IQR) (mg/dL)	0.80 (0.91)	0.71 (0.38)	< 0.001
Median BUN (IQR) (mg/dL)	21 (20.8)	13 (9.8)	< 0.001
Mean Na+ (SD) (mmol/L)	139.2 (5.28)	136.8 (4.53)	< 0.001
Mean TCO2 (SD) (mmol/L)	26.8 (4.9)	24.8 (3.68)	0.001
Median PLT (IQR) (cells/µL)	289 (300.5)	352.5 (344.8)	< 0.001

*BUN* blood urea nitrogen, *HCT* hematocrit, *HGB* hemoglobin, *ICU* intensive care unit, *F* female, *GCS* Glascow Coma Score, *IQR* interquartile range, *LOS* length-of-stay, *M* male, MODS multiple organ dysfunction score, *N/A* not applicable, *Na*+ ionized sodium, *NS* not significant, *PLT* platelet count, *SD* standard deviation, *SOFA* sequential organ failure assessment score, *TBSA* total body surface area, *TCO2* total CO2, and *WBC* white blood cell count.

### Generalization performance of ML algorithms produced by traditional ML versus the MILO approach

Traditional ML approach required 400 h to train and test various models and utilizing manual interventions and discussions, while MILO automatically completed the same programming tasks plus additional comparisons in 20 h.

Table 4 compares the generalization performance for the top five performing ML models on each given algorithm produced by our traditional ML programming approach versus MILO’s Auto-ML approach which included DNN, LR, NB, *k-*NN, SVM, RF, and GBM methods. For ML model development using both traditional and MILO techniques, our heuristic approach employed a balanced training dataset of 250 culturing events that resulted in sepsis and 250 that did not result in sepsis pulled from the 704 events derived from the 211 patient dataset. The remaining 204 culturing events which represented the study population’s sepsis prevalence (48 sepsis and 156 non-sepsis), were then used for the final validation/generalization of each of the ML models.

**Table 4:** Machine learning algorithm performance for the top 5 models identified by traditional programming versus MILO.

Method	Accuracy (95% CI)	AUROC (95% CI)*	Sensitivity (95% CI)	Specificity (95% CI)	Features
**A. Traditional programming**					
Logistic regression	86 (80–90)	0.96 (0.88–1.00)	98 (89–100)	82 (75–88)	16^a^
Deep neural network	81 (75–86)	0.96 (0.85–1.00)	94 (83–99)	77 (70–83)	10^b^
k-nearest neighbor	81 (75–86)	0.92 (0.84–1.00)	98 (89–100)	76 (68–82)	10^b^
Support vector machine	85 (79–89)	0.97 (0.86–1.00)	98 (89–100)	81 (74–87)	14^c^
Random forest	79 (73–85)	0.92 (0.84–1.00)	94 (83–99)	75 (67–82)	10^b^
**B. MILO**					
k-nearest neighbor	90 (85–94)	0.96 (0.85–1.00)	96 (86–99)	88 (82–93)	5^e^
Logistic regression	87 (81–91)	0.95 (0.83–1.00)	98 (89–100)	83 (77–89)	23^f^
Naïve bayes	89 (84–93)	0.95 (0.84–1.00)	94 (83–99)	87 (81–92)	11^d^
Random forest	84 (79–89)	0.94 (0.84–1.00)	96 (86–99)	81 (74–87)	23^f^
Deep neural network	84 (79–89)	0.95 (0.85–1.00)	100 (93–100)	80 (72–86)	17^ g^
Support vector machine	86 (80–90)	0.97 (0.87–1.00)	98 (89–100)	82 (75–88)	11^d^
Gradient boosting machine	81 (75–86)	0.94 (0.88–1.00)	96 (86–99)	76 (69–83)	5^e^

*BUN* blood urea nitrogen, *CI* confidence interval, *CVP* central venous pressure, *DBP* diastolic blood pressure, *GCS* Glascow Coma Score, *HCT* hematocrit, *HGB* hemoglobin, *HR* heart rate, *MAP* mean arterial pressure, *MODS* multiple organ dysfunction score, *PLT* platelet count, *RR* respiratory rate, *SBP* systolic blood pressure, *SO2* oxygen saturation, *TCO2* total CO2, and *WBC* white blood cell count.

*Area under the ROC curves are reported in fractions.

^a^MAP, RR, body temperature, GCS, WBC, HGB, HCT, PLT, Na+ , K+ , BUN, creatinine, BUN/creatinine, glucose, TCO2, and MODS.

^b^Body temperature, WBC, HGB, HCT, Na+ , K+ , BUN, creatinine, BUN/creatinine, and TCO2.

^c^RR, body temperature, GCS, WBC, HGB, HCT, PLT, Na+ , K+ , BUN, creatinine, BUN/creatinine, TCO2, and MODS.

^d^SBP, MAP, HR, TEMP, HCT, Na+ , K+ , BUN, BUN/creatinine, anion gap, and TCO2.

^e^HR, body temperature, HGB, BUN, and TCO2.

^f^SBP, DBP, MAP, CVP, RR, HR, body temperature, GCS, SO2, WBC, HGB, HCT, PLT, Na+ , K+ , Cl−, anion gap, BUN, creatinine, BUN/creatinine, glucose, TCO2, and MODS.

^g^MAP, HR, RR, TEMP, WBC, HGB, HCT, PLT, Na+ , K+ , BUN, creatinine, BUN/creatinine, glucose, anion gap, TCO2, and MODS.

First, using our traditional ML approach, comparisons (i.e., 4,540 models times 11 categories) were made for the generated 49,940 ML models. Within this approach, the best performing ML model used LR with 16 features (i.e., mean arterial pressure, respiratory rate, body temperature, GCS, WBC, HGB, HCT, PLT, Na+ , K+ , BUN, plasma creatinine, glucose, TCO2, and MODS) with an accuracy of 86% and an area under the ROC curve of 0.96.

**Figure 3:**
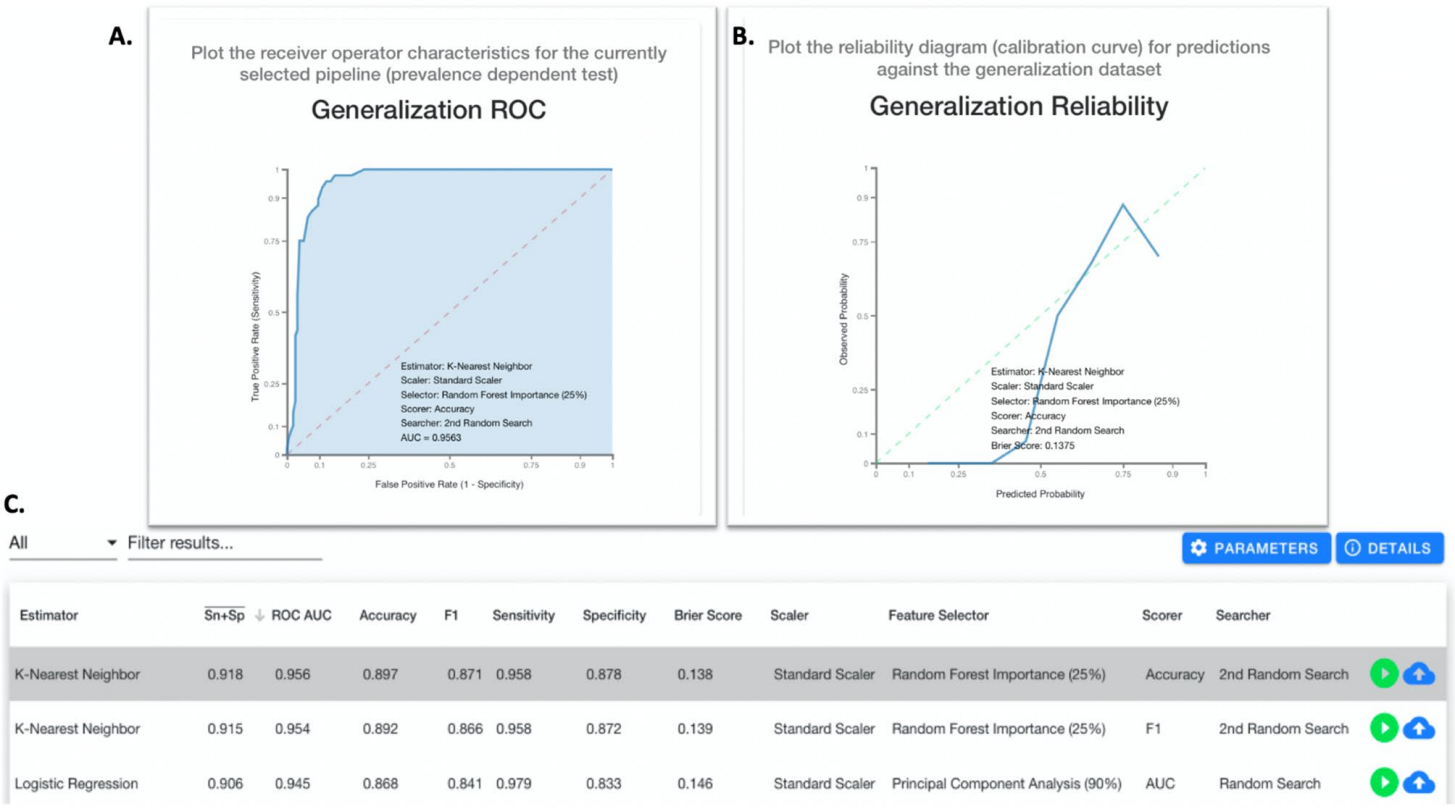
MILO ROC for optimal ML model: screenshot of the optimal machine learning (ML) model generated by MILO based on logistic regression. (**A**) is the MILO read out for the receiver operator characteristic (ROC) curve using the selected ML model (i.e., logistic regression). (**B**) is the generalization/reliability plot for the selected ML model. (**C**) is the filtered list of ML models displaying other parameter such as average sensitivity and specificity (Sn + Sp “bar”), area under the curve (AUC) for the ROC analysis, F1 score, binary sensitivity and specificity, Brier Score, scaler used, feature selector used, scorer used, and searcher used.

For MILO, 345,330 model comparisons were automatically produced for the ML models using the same training-generalization split for the data (Supplemental Data Table S2). Based on the MILO approach, a *k*-NN model using heart rate, body temperature, HGB, BUN, and TCO2 as features was found to be the best performer. The MILO *k*-NN accuracy was found to be 89.7% with an area under the ROC curve of 0.96 (95% CI 0.85–1.0). Clinical sensitivity and specificity were 95.8% and 87.8% respectively (Fig. 3). When using only ABA Consensus Guidelines consisting of body temperature, respiratory rate, heart rate, PLT, and glucose, the optimal model produced by MILO achieved an accuracy of 67.1% with an area under the ROC curve of 0.76 (95% CI 0.68–0.82) using the RF approach. Clinical sensitivity and specificity were 75.0% and 65.7% respectively. In contrast, applying Sepsis-3 criteria with ML exhibited an optimized accuracy of 74.5% using NB with an area under the ROC curve of 0.55 (95% CI 0.50–0.81), and sensitivity of 61.2% and specificity of 55.1%.

## Discussion

Burn sepsis exhibits high mortality attributed, in part, to delayed recognition of infection. Studies have indicated traditional sepsis criteria are not suitable for this unique population^1^. Recent investigations suggest ML may be able to identify unique pathologic patterns not recognized by the “human eye” and enhance the performance of traditional biomarkers for certain diseases (e.g., acute kidney injury)^8,11^. In this article, we report the use of ML for predicting sepsis in this unique high-risk burn population. Study data was based on a unique ABA sponsored multicenter randomized controlled trial that ensured complete and high-quality data for the entirety of each patient’s ICU stay.

Burn sepsis recognition is challenged by the underlying hypermetabolic and inflammatory response that persists for days or months following injury^1^. Using a combination of heart rate, body temperature, HGB, BUN, and TCO2, *k*-NN was able to predict burn sepsis with high accuracy, sensitivity, and specificity—exceeding the performance of both the ABA Consensus Guidelines and Sepsis-3 criteria reported in literature. These findings are clinically significant, since this is the first time burn sepsis has been detected with this level of accuracy, sensitivity, and specificity. Interestingly, PLT, which is often included in burn sepsis criteria was excluded in our optimal ML algorithm. This observation may be due to PLT representing a late-stage indicator of burn sepsis, while inclusion of HGB and TCO2 may be attributed to hematological derangements and acid–base/electrolyte disturbances encountered during sepsis^13–15^. Interestingly, when MILO was limited to the ABA Consensus Guidelines or Sepsis-3 criteria, the optimal model performed marginally better than traditional statistics. These findings highlight the capacity for ML, and especially MILO’s ability to run an exhaustive search of models and across all collected variables to identify clinically significant patterns not observed by the human eye or by traditional statistics.

In the end, the ABA Consensus Guidelines provides a framework to improve burn sepsis recognition, however its clinical performance remains limited with an area under the ROC curve of 0.62 based on current literature^16^. Studies by Mann-Salinas et al*.* proposed novel predictors that could outperform the ABA Consensus Guidelines with an area under the ROC curve of 0.78. We were able to improve predictive performance using traditional statistics in our study and achieve an area under the ROC curve of 0.88. Sepsis-3 has also been investigated, however its heavy reliance on the SOFA score remains controversial in multiple populations including in burned patients. It must be noted that a new study recently suggested Sepsis-3 may be superior to ABA Consensus Guidelines^5^, however, performance appears to still lag behind AI/ML models produced in this study.

Despite the promising performance of various ML algorithms reported in literature for in vitro diagnostic applications^9^, the accessibility of ML in healthcare remains limited to facilities employing experienced programmers and data science experts^17^. Unique to this study, we employed an automated ML application, called MILO. MILO eliminates these limitations and improves the accessibility and feasibility of ML-based data sciences, and more importantly, helps identify the optimal ML models in an unbiased and transparent way. No assumptions are made on a given dataset using MILO and programming expertise is not necessary. The benefits of using an automated ML platform not only accelerates development of new predictive models (400 vs. 20 h), but also identifies the best performing algorithm after running a very large feature and ML model combinations. Ultimately, MILO serves to limit selection and method bias to make ML more accessible to the general public.

Limitations of this study include its retrospective nature and the use of protocolized clinical trial data from another study. In particular, clinical trial data is more controlled compared to what is encountered on a routine basis, however, the data does offer the benefit of having been vetted for accuracy and completeness. Reproducibility of AI is also a concern due to differences in populations, test methodology, differences in test practices across institutions/disciplines, and ML methods^9,18^. Thus, the generalizability of the data is limited to this retrospective dataset, therefore additional studies are needed to further verify performance and assess how these ML algorithms perform under real-world conditions prior to any clinical implementation.

## Conclusions

Sepsis contributes to high mortality in the severely burned patient population. Existing sepsis criteria are not well suited to differentiate between the host response to infection versus burn injury. Machine learning offers unique opportunities to exploit and enable sophisticated computer-based pattern recognition tools to predict sepsis in the burn patient population. Traditional methods for creating ML is both time consuming and relies on experienced programmers to work through a large permutation of features across a range of models. The deployment of MILO not only accelerates the development of ML models, but quickly helps identify optimal features and algorithms for burn sepsis prediction. Future multicenter studies are needed to refine the performance and confirm generalizability of ML models. Next steps include deploying the MILO burn sepsis algorithm at our institution to observationally test its accuracy when using prospective data.

## Supplementary information

Supplementary Information
