# Supplementary material for: Novel application of an automated-machine learning development tool for predicting burn sepsis: proof of concept
Source: Sci Rep. 2020 Jul 23;10:12354. doi: 10.1038/s41598-020-69433-w (PMC7378181; doi:10.1038/s41598-020-69433-w)
Supplement: Supplementary file 1 — Supplementary Information [file 41598_2020_69433_MOESM1_ESM.pdf]

SCIENTIFIC REPORTS – SUPPLEMENTAL DATA

NOVEL APPLICATION OF AN AUTOMATED-MACHINE LEARNING DEVELOPMENT TOOL FOR PREDICTING BURN SEPSIS: PROOF OF CONCEPT

Nam K. Tran, PhD, HCLD (ABB), FAACC[1*], Samer Albahra, MD[1];
Tam N. Pham, MD, FACS[2]; James H. Holmes IV, MD, FACS[3];
David Greenhalgh, MD, FACS[4]; Tina L. Palmieri, MD, FACS, FCCM[4];
Jeffery Wajda, DO, MS, FACEP[5]; and Hooman H. Rashidi, MD, FASCP[1*]

Figure S1. Flow Diagram

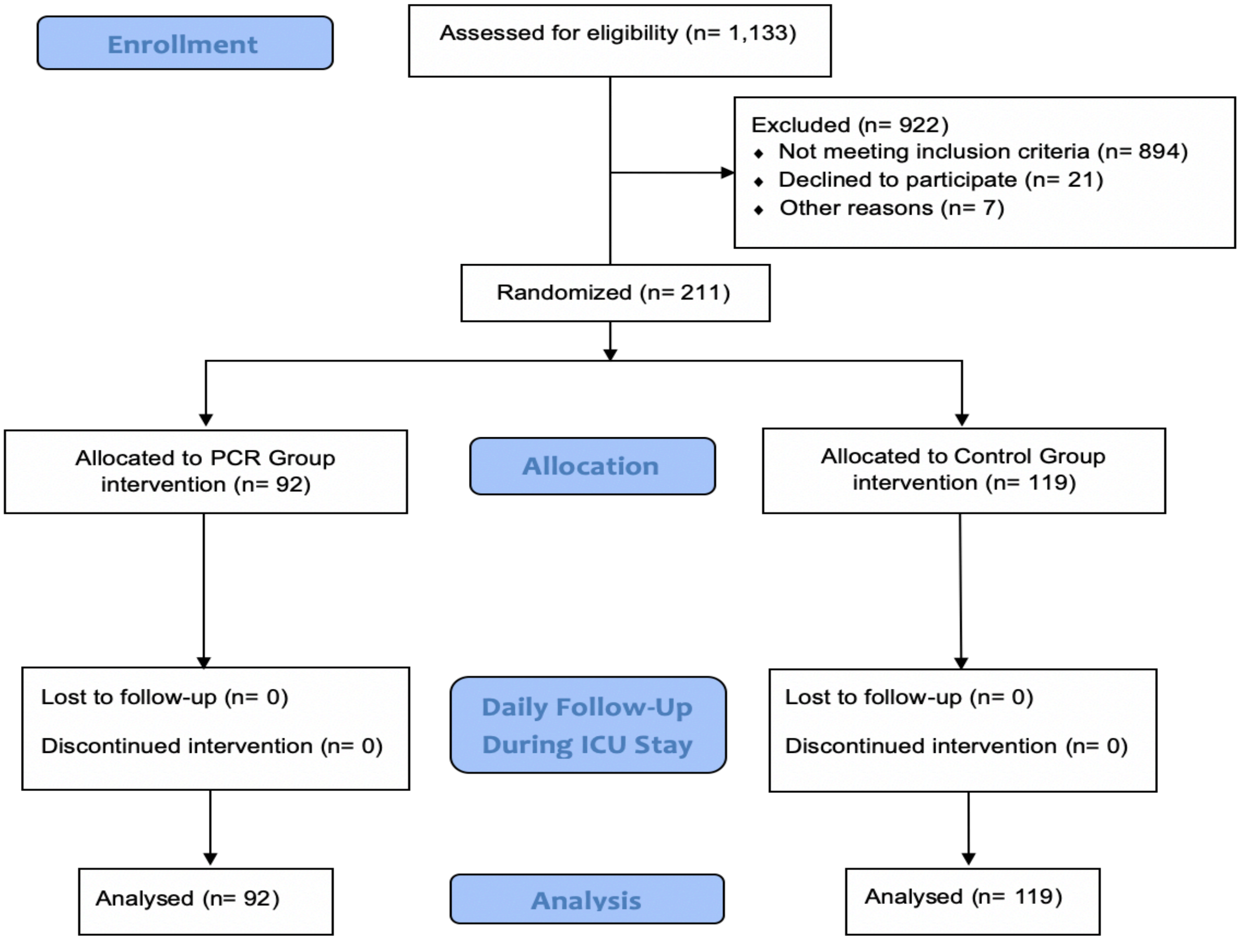

Table S1. Comparison of our Non-automated versus Automated machine learning

	**Our Non-Automated ML Approach**	**Our Automated ML (MILO) Approach**
**Algorithms***	KNN, LR, SVM, DNN and RF	KNN, LR, SVM, DNN, RF, NB and GBM
**Scaler(s) used**	Standard scaler	standard scaler, Min/max, and no scaler
**Feature Selector and/or transformers used**	ANOVA F Value Select percentile (10% increments)	• ANOVA F Value Select percentile (25% increments)<br>• Random Forest Feature Importances (25% increments) and<br>• principal component analysis
**Hyperparameter Searchers**	Grid search	- Grid search and<br>- Random Search x2
**Scorer(s) used in the training/initial validation phase**	Accuracy	• Accuracy<br>• ROC-AUC<br>• F1
**Model Assessments**	Generalization assessment performed on best accuracy model	Generalization assessment on all pipelines

Table S2. Number of Models Generated by MILO for Sepsis Data

Algorithm	Search	Scalers	Feature Selectors	(Hyperparameter Search count x k-fold of 10) + 3 scorers	Total
GBM	Grid	3	9	(30 x 10) + 3	8181
GBM	Random 1	3	9	(100 x 10) + 3	27081
GBM	Random 2	3	9	(100 x 10) + 3	27081
KNN	Grid	3	9	(56 x10) + 3	15201
KNN	Random 1	3	9	(100 x 10) + 3	27081
KNN	Random 2	3	9	(100 x 10) + 3	27081
LR	Grid	3	9	(27 x 10) + 3	7371
LR	Random 1	3	9	(100 x 10) + 3	27081
LR	Random 2	3	9	(100 x 10) + 3	27081
DNN	Grid	3	9	(72 x 10) + 3	19521
DNN	Random 1	3	9	(100 x 10) + 3	27081
DNN	Random 2	3	9	(100 x 10) + 3	27081
NB	Grid	3	9	(1 x 10) + 3	351
NB	Random 1	3	9	(1 x10) + 3	351
NB	Random 2	3	9	(1 x10) + 3	351
RF	Grid	3	9	(45 x10) + 3	12231
RF	Random 1	3	9	(100 x 10) + 3	27081
RF	Random 2	3	9	(100 x 10) + 3	27081
SVM	Grid	2	9	(40 x10) + 3	7254
SVM	Random 1	2	9	(10 x 10) + 3	1854
SVM	Random 2	2	9	(10 x 10) + 3	1854
					345330
